# Gastric Lipase Secretion in Children with Gastritis

**DOI:** 10.3390/nu5082924

**Published:** 2013-07-29

**Authors:** Przemyslaw J. Tomasik, Andrzej Wędrychowicz, Iwona Rogatko, Andrzej Zając, Krzysztof Fyderek, Krystyna Sztefko

**Affiliations:** 1 Department of Clinical Biochemistry, Collegium Medicum, Jagiellonian University, Wielicka St. 265, Krakow 30-663, Poland; E-Mails: iwonarogatko@poczta.onet.pl (I.R.); sekretariatbiochem@op.pl (K.S.); 2 Department of Pediatrics, Gastroenterology and Nutrition, Collegium Medicum, Jagiellonian University, Wielicka St. 265, Krakow 30-663, Poland; E-Mails: awedrycho@cm-uj.krakow.pl (A.W.); fkrzy@mp.pl (K.F.); 3 Department of Pediatric Surgery, Collegium Medicum, Jagiellonian University, Wielicka St. 265, Krakow 30-663, Poland; E-Mail: zycek@hotmail.com

**Keywords:** CCK, gastritis, GIP, GLP-1, *Helicobacter pylori*, human gastric lipase, non-HP gastritis

## Abstract

Gastric lipase is one of the prepancreatic lipases found in some mammalian species and in humans. Our knowledge of the hormonal regulation of gastric lipase secretion in children and adolescents is still very limited. The aim of this study was to compare the activity of human gastric lipase (HGL) in gastric juice in healthy adolescents and in patients with gastritis. The adolescents were allocated to three groups: the first including patients with *Helicobacter pylori* gastritis (HPG; *n* = 10), the second including patients with superficial gastritis caused by pathogens other than *H. pylori* (non-HPG; *n* = 14) and the control group including healthy adolescents (*n* = 14). Activity of HGL was measured in gastric juice collected during endoscopy. Plasma concentrations of cholecystokinin (CCK), glucagon-like peptide-1 (GLP-1) and glucose-dependent insulinotropic peptide (GIP) were measured in all adolescents. Activity of HGL in the non-HPG group was significantly lower than in the HPG group (*p* < 0.005) and the control group (*p* < 0.005). Mean plasma GIP levels in the control group were lower than in the non-HPG group (*p* < 0.003) and the HPG group (*p* < 0.01). We conclude that the regulation of HGL secretion by GLP-1 and CCK is altered in patients with gastritis. Moreover, GIP is a potent controller of HGL activity, both in healthy subjects and in patients with gastritis.

## 1. Introduction

Gastric lipase is one of the mammalian preduodenal lipases (also termed prepancreatic lipases) [1,2,3]. Immunochemical studies revealed that human gastric lipase (HGL) is secreted by the chief (zymogen) cells of gastric mucosa [4]. HGL takes part in digestion of fats, and it accounts for approximately 40% of preduodenal lipolysis. In newborns, the activity of pancreatic lipase is low, and therefore, the bile salt stimulated lipase (BSSL), present in breast milk, and HGL are important for digestion of fats in this period of life [5]. In older children and in adults, HGL may also be important. It has been suggested that products of gastric hydrolysis of dietary triacylglycerol may promote lipolysis and regulate intestinal function [6]. Supplementation with acid-stable gastric lipase seems to be one of the potential therapies of pancreatic insufficiency [7,8]. Supplementation with exogenous gastric lipase has no adverse effects typical for supplementation with pancreatic lipase, like fibrosing colonopathy [9,10]. A recent study using an animal model suggests that inhibition of gastric lipase might be used to treat obesity and improve lipid profile [11]. It has been suggested that in healthy subjects, the regulatory peptides, like glucagon-like peptide-1 (GLP-1) and cholecystokinin (CCK), may influence secretion and/or activity of HGL [12]. Another peptide taking part in the regulation of the upper gastrointestinal function is glucose-dependent insulinotropic peptide (GIP), but we have not identified any data on the effects of GIP on gastric lipase secretion. Moreover, little is known about the regulation of HGL secretion in patients with gastric disorders, including gastritis, which is the most common gastric condition. Therefore, the aim of our study was to evaluate the activity of HGL in gastric juice collected from adolescents with superficial gastritis caused by *Helicobacter pylori* and with superficial gastritis caused by other factors, as well as from healthy subjects. In addition, plasma concentrations of major enterogastrones, GLP-1, CCK and GIP, which are potential regulators of HGL secretion/activity, were measured in all the adolescents taking part in the study.

## 2. Experimental Section

### 2.1. Groups of Patients

Thirty eight adolescents aged 16 ± 3 years hospitalized in the University Children’s Hospital of Krakow were included in the study. The protocol was approved by the local Bioethics Committee. Written informed consent was obtained from all patients and their guardians prior to the participation in the study.

The adolescents were allocated to three groups based on the results of gastroscopy, histology of the biopsy material and urease test for *H. pylori*. The group of patients with *H. pylori* gastritis (HPG, *n* = 10) included the patients with gastritis, in whom the diagnosis was based on macroscopic findings in gastroscopy and confirmed by histological methods, including a positive urease test and positive histological assessment for *H. pylori* using Giemsa stain. The group of patients with gastritis caused by factors other than *H. pylori* (non-HPG, *n* = 14) included the patients with superficial gastritis, in whom the diagnosis was based on macroscopic findings in gastroscopy and confirmed by histological methods, including a negative urease test and negative histological assessment for *H. pylori* using Giemsa stain. The control group (CG, *n* = 14) included adolescents with no macroscopic abnormalities on gastroscopy and with normal histology of the biopsy samples.

Height and body weight measurements were performed by an anthropometrist. The Body Mass Index (BMI) and BMI percentiles were calculated using online BMI calculators for patients ≤20 years of age.

### 2.2. Gastric Juice Collection Method

For 7 days before the gastroscopy, the patients did not receive any treatment that may have stimulated or inhibited the production of gastric juice. Gastric juice was collected during routine gastroscopy using the trapping method. Immediately after the gastroscope was introduced into the stomach, a 3 mL sample of gastric juice was suctioned via the auxiliary channel of the gastroscope and collected in a sterile vial. The samples were then centrifuged at 5000× *g* for 15 min at 4 °C to remove cell debris and other contaminations. Subsequently, the pH of the supernatant was measured, and the samples were frozen at −20 °C until assayed.

### 2.3. Laboratory Tests

#### 2.3.1. Measurements of pH

Measurements of the pH of the collected samples were performed using the InLab Micro electrode (Mettler Toledo; Schwarzenbach, Switzerland) for microsamples, connected to the Seven easy pH-meter (Mettler Toledo, Schwarzenbach, Switzerland).

#### 2.3.2. Measurements of Human Gastric Lipase Activity in Gastric Juice

HGL activity in gastric juice was determined using the colorimetric method with 10 nM methanol solution of *p*-nitrophenyl caprylate. Twenty microliters of 10 mM *p*-nitrophenyl caprylate solution was added to 1 mL of centrifuged gastric juice. Absorbance was measured continuously at controlled conditions of pH 4.5 and 25 °C using a spectrophotometer with a thermostatic cuvette and continuous absorbance reading at 420 nm in a 1-cm path-length cell. A control assay without the enzyme was performed for each HGL activity measurement under the same conditions. The obtained value, corresponding to the autohydrolysis of the substrate, was subtracted from the calculation of HGL activity in samples. The amount of *p*-nitrophenol formed from *p*-nitrophenyl caprylate was calculated to assess the activity of HGL (1 μmol of *p*-nitrophenyl corresponding to one unit (U) of enzyme activity).

#### 2.3.3. Measurements of Peptide Concentrations in Blood

Fasting blood samples for hormone measurements were collected to chilled glass tubes containing EDTA (4 mg) and aprotinin (Sigma-Aldrich, St. Louis, MO, USA) (0.2 TIU (trypsin inhibitor unit)) in the morning, shortly before the gastroscopy. Immediately after the sampling, the tubes were transported in ice to the laboratory. Blood was centrifuged at +4 °C for 10 min at 3000× *g*. Plasma samples were stored at −20 °C until assayed. Concentrations of peptides were determined by radioimmunoassay (RIA) using commercial kits—CCK (Eurodiagnostica, Sweden), GIP and GLP-1 (7–37) (Phoenix Pharmaceuticals, Inc., Burlingame, CA, USA). The extraction of CCK from plasma was carried out according to the following procedure: plasma (1 mL) was mixed with 96% ethanol (2 mL) followed by 20 min centrifugation at +4 °C and 7000× *g*. The supernatant was evaporated in the stream of the nitrogen. The dry residue was dissolved in the RIA buffer immediately before the assay. Plasma samples for GIP and GLP-1 assays were extracted on the Sep-pak C-18 columns (Waters Corporation, Milford, MA, USA). Prior to the extraction, the plasma samples were acidified with 1% TFA (Sigma-Aldrich, St. Louis, MO, USA) (1:1, v/v), followed by 20 min centrifugation at +4 °C and 7000× *g*. The supernatant was applied on the column and washed three times with 3 mL 1% TFA solution. The peptides were then eluted off with 60% acetonitrile solution (3 mL) (Spectrosol, England) in 1% TFA. The eluate was evaporated under nitrogen at 40 °C. The dry residue was dissolved in the appropriate RIA buffer immediately before the assay. The recovery test using the control samples containing the known amounts of peptides were 92% ± 3% for CCK, 84% ± 4% for GIP and 84% ± 4% for GLP-1.

### 2.4. Statistical Analysis

All the results are reported as mean values ± SD. Statistical analysis was carried out using the Student’s *t*-test. Correlation analysis was performed using the Statistica 10 (Statsoft) and Excel (Microsoft Office Home and Student 2010) software. A *p*-value of 0.05 was considered statistically significant.

## 3. Results

### 3.1. Anthropometric Measurements

There were no significant differences between the study groups with respect to age, body weight and BMI.

### 3.2. pH

We found no differences of acidity (pH) of the gastric juice between the study groups (non-HPG 1.55 ± 0.27; HPG 1.53 ± 0.20; CG 1.5 ± 0.25).

### 3.3. HGL Activity

**Figure 1:**
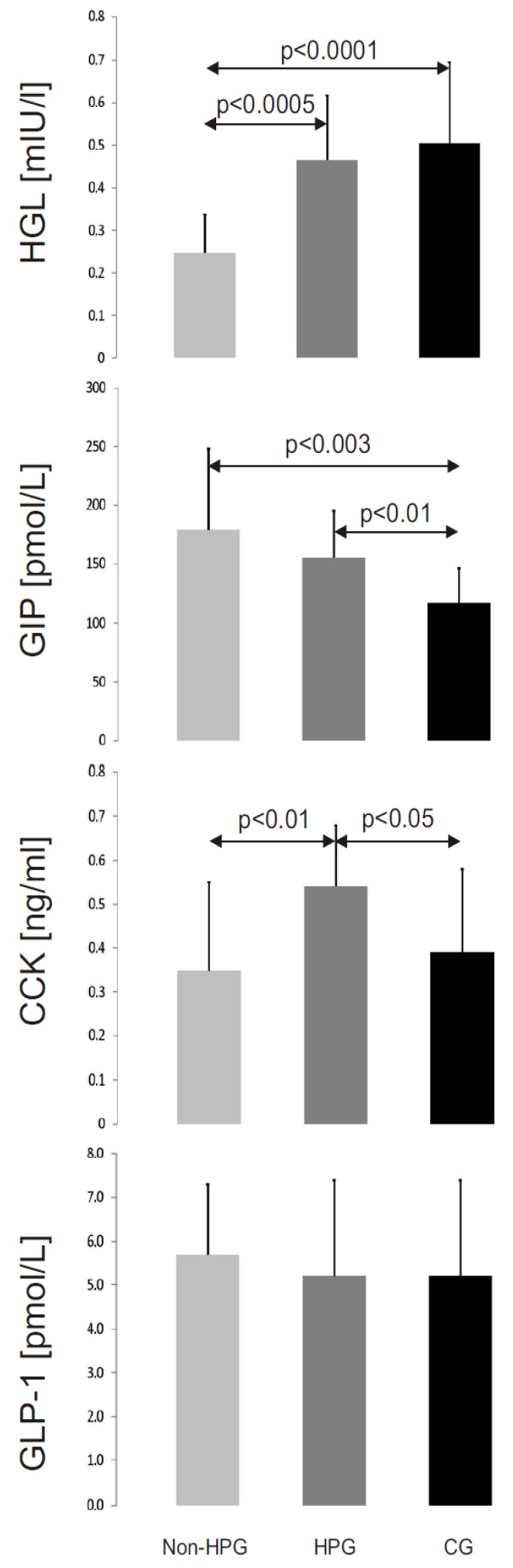
The mean activity of human gastric lipase (HGL) and mean concentrations of cholecystokinin (CCK), glucagon-like peptide-1 (GLP-1) and glucose-dependent insulinotropic peptide (GIP) in the study groups (non-HPG—non-*Helicobacter pylori* gastritis group, HPG—*Helicobacter pylori* gastritis group, CG—control group).

The mean activity of HGL in gastric juice in the non-HPG group (0.247 ± 0.09 mU/L ) was significantly lower than in the two remaining groups (HPG—0.466 ± 0.151 mU/L, *p* < 0.0005; CG—0.504 ± 0.191 mU/L, *p* < 0.0001; Figure 1). The differences of HGL activity between CG and HPG groups were not significant (*p* = 0.33).

### 3.4. Peptide Concentrations

Mean plasma levels of GIP were lower in the control group (117 ± 29 pmol/L) than in the non-HPG group (179 ± 69 pmol/L, *p* < 0.003) and the HPG group (155 ± 40 pmol/L, *p* < 0.01) (Figure 1). No statistically significant differences of GIP concentrations between the non-HPG and HPG groups were observed (*p* = 0.21). Mean plasma concentrations of CCK were highest in the HPG group (0.54 ± 0.14 ng/mL). They were significantly higher than in the non-HPG group (0.35 ± 0.2 ng/mL, *p* < 0.01) and the control group (0.39 ± 0.19 ng/mL, *p* < 0.05) (Figure 1). The differences of CCK concentrations between the CG and non-HPG groups were not significant (*p* = 0.34). Mean plasma concentrations of GLP-1 were similar in all study groups (non-HPG—5.7 ± 1.6 pmol/L; HPG and CG—5.2 ± 2.2 pmol/L; *p-*values for statistical comparisons: CG *vs*. non-HPG, *p* = 0.23; CG *vs*. HPG, *p* = 0.33; non-HPG *vs*. HPG, *p* = 0.45; Figure 1).

### 3.5. Correlations

**Figure 2:**
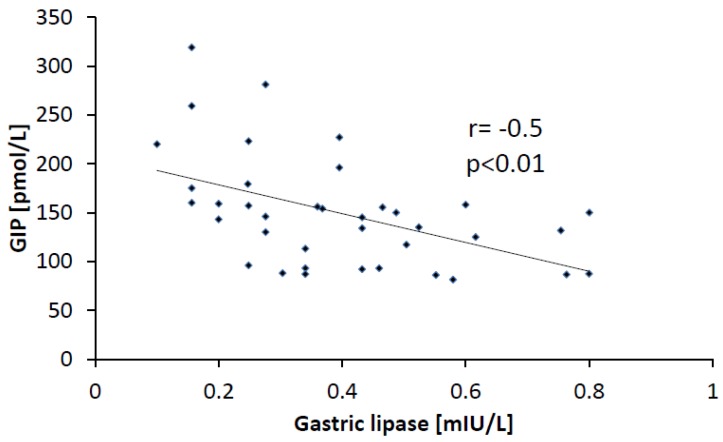
Correlation between activity of HGL and concentration of GIP in all studied patients.

The analysis of pooled results performed in all three groups revealed significant correlation between the activity of HGL and the plasma concentration of GIP (*r* = −0.50; *p* < 0.01) (Figure 2). Similarly, statistically significant correlation was observed between the non-HPG group (*r* = −0.69; *p* < 0.001) and the control group (*r* = −0.39, *p* < 0.05). In the HPG group, a negative correlation was also found, but it did not reach statistical significance. Significant positive correlation between HGL activity and CCK concentrations was also observed in the control group (*r* = 0.40; *p* < 0.05). No other statistically significant correlations between the activity of HGL and the concentrations of the measured peptides were found within the study groups, as well as in the whole study population.

## 4. Discussion

The only available data on the activity of HGL in patients with gastritis were published by Sarles *et al*. in 1992 [13]. The authors studied a group of 55 children aged from zero to 17 years, including only nine patients with gastritis. Unfortunately, the details of the age of the patients and the etiology and severity of gastritis were not provided in the publication. Sarles *et al*. reported that in seven children with gastritis, the activity of HGL was normal, while in the two remaining children, only a trace HGL activity was found. They concluded that the activity of HGL is only rarely reduced in patients with gastritis [13]. In our study, the age of the patients was in a similar range (adolescents), and all of them had gastritis. Moreover, all our patients were tested for *H. pylori* infection. Therefore, our results are only partially comparable to the data published by Sarles *et al*. [13]. We have found that in the patients with *H. pylori* gastritis, the mean activity of HGL in gastric juice was similar to that found in healthy subjects. However, the activity of HGL in patients with non-*H. pylori* gastritis was significantly lower than in the control group (*p* < 0.0001; Figure 1).

Several factors may influence the secretion of HGL. Firstly, the differences in the acidity of gastric juice should be taken into account. HGL is an acid-stable lipase, with the optimum activity at the pH values of approximately five [14]. However, in our study, pH values had no effect on the activity of HGL, because in all study groups, the pH of gastric juice was similar. Another explanation could be duodenal reflux, which may cause the displacement of bile into gastric contents. Reflux was observed in more than half of the patients with gastritis caused by *H. pylori*. Contamination of gastric contents with bile salts should decrease the activity of HGL, as taurocholate is known to be an inhibitor of HGL [15,16]. However, conflicting data were published in the literature, showing that low levels of taurocholate may actually increase the activity of HGL [17]. Another explanation of this phenomenon may be an alteration of endogenous control mechanisms of HGL secretion. In healthy adults, gastrin increases the secretion of HGL [18]. Wojdemann *et al*. and Borovicka *et al*. observed that GLP-1 and CCK inhibited the secretion of HGL [12,19,20]. They found that an intravenous infusion of GLP-1 imitating the postprandial levels of this peptide reduced both secretion and activity of HGL in humans [19]. On the other hand, an infusion of loxiglumide (CCK-A receptor antagonist) increased the secretion of HGL [20]. In our study, the concentrations of CCK, GLP-1 (amide) and GIP were measured. We found no differences in GLP-1 concentrations between the study groups and no correlation between the levels of GLP-1 and the activity of HGL. Surprisingly, the concentrations of CCK, which was described by Borovicka *et al*. as an inhibitor of HGL secretion, as well as a stimulator of pancreatic lipase secretion, showed positive correlation with the activity of HGL [20]. This suggests that either the regulation of HGL secretion by GLP-1 and CCK is abnormal in gastritis or there exists another factor with higher potential for regulation of the secretion and activity of HGL. The small size of our study may be a source of bias, though it must be noted that Borovicka studied six subjects only [20]. To date, GIP has not been analyzed as a factor influencing the secretion or activity of HGL. However, GIP is a known activator of lipoprotein and pancreatic lipases [21,22]. The negative correlation between the concentrations of GIP and the activity of HGL in the whole study population suggests that GIP has an inhibitory effect on HGL secretion similar to other enterogastrones (CCK and GLP-1). In addition, GIP could be one of the main regulators of the secretion and activity of HGL in healthy subjects and in patients with gastritis.

The role of HGL in the digestion of lipids is significant, due to its activity in a wide range of pH values. Petersen *et al*. observed lipolytic activity of HGL in solutions of high acidity (pH 2.8) [23]. HGL is also active in the duodenum, where it is responsible for hydrolysis of 7.5% of triglycerides [24]. Vaquero *et al*. showed in an animal model that inhibition of gastric lipase caused a moderate reduction of fat absorption, resulting in reduction of weight gain, as well as triglyceride and cholesterol levels [11]. These data suggest that HGL is a potential target for the treatment of obesity, and therefore, the results of our study may have implications for future research on the treatment of obesity.

## 5. Conclusions

1. The activity of HGL is reduced in patients with non-*H. pylori* gastritis.
2. Regulation of HGL activity by GLP-1 and CCK is abnormal in patients with gastritis.
3. GIP is a potent regulator of HGL activity.
